# Role of a Modified Urothelium Immune Prognostic Index in Patients With Metastatic Urothelial Carcinoma Treated With Anti–PD-1/PD-L1–Based Therapy

**DOI:** 10.3389/fmolb.2021.621883

**Published:** 2021-08-12

**Authors:** Haifeng Li, Xin An, Riqing Huang, Lu Li, Chengbiao Chu, Wei Yang, Zike Qin, Zhuowei Liu, Fangjian Zhou, Cong Xue, Yanxia Shi

**Affiliations:** ^1^ State Key Laboratory of Oncology in South China, Collaborative Innovation Center for Cancer Medicine, Sun Yat-Sen University Cancer Center, Guangzhou, China; ^2^ Department of Medical Oncology, Sun Yat-sen University Cancer Center, Guangzhou, China; ^3^ Department of Pathology, Sun Yat-sen University Cancer Center, Guangzhou, China; ^4^ Department of Urology, Sun Yat-sen University Cancer Center, Guangzhou, China

**Keywords:** PD-1 inhibitor, LDH, NLR, urothelial carcinoma, mUIPI

## Abstract

**Introduction:** The use of antibodies against programmed death receptor-1 (PD-1) and its ligand (PD-L1) has improved survival in metastatic urothelial carcinoma (mUC) patients. However, reliable and convenient biomarkers of early responses and outcomes are still lacking.

**Materials and Methods:** We retrospectively screened mUC patients who received anti–PD-1/PD-L1–based therapy at our institute. A modified urothelium immune prognostic index (mUIPI) based on the neutrophil-to-lymphocyte ratio (NLR) and lactate dehydrogenase (LDH) was developed to characterize the three groups as good, intermediate, and poor mUIPI. Major observations were progression-free survival (PFS), overall survival (OS), and disease control rate (DCR).

**Results:** We identified 52 mUC patients with a median follow-up time of 29.8 months (95% CI, 26.3–53.2). Low NLR was with improved PFS and OS (hazard ratio [HR], 0.40, 95% CI, 0.18–0.92; HR, 0.27, 95% CI, 0.11–0.69, respectively). Normal LDH was associated with improved PFS but not OS (HR, 0.22, 95% CI, 0.10–0.52; HR, 0.86, 95% CI, 0.34–2.13, respectively). The median PFS for the poor, intermediate, and good mUIPI groups was 1.97 months (95% CI, 1.15 to NR), 3.48 months (95% CI, 1.58 to NR), and 14.52 months (95% CI, 5.75 to NR), respectively (*p* < 0.001). The median OS for the poor, intermediate, and good mUIPI was 12.82, 18.11, and 34.87 months, respectively (*p* = 0.28). A good mUIPI was associated with a higher DCR compared to intermediate and poor mUIPI (odds ratio [OR] 7.58, 95% CI, 1.73–43.69; OR, 6.49, 95% CI, 0.14–295.42, respectively). In the subgroup analysis, a good mUIPI was associated with improved PFS in the subgroups of male patients and patients with low urinary tract primary tumors, liver metastases, non–first-line treatment, and monotherapy.

**Conclusions:** mUIPI predicts early responses in mUC patients who received anti–PD-1/PD-L1–based therapy.

## Introduction

Urothelial cancer (UC) is one of the most common malignant tumors of the urinary system in the world (Siegel et al., 2019). In China, UC incidence is 6.61/100,000, ranking sixth of all malignant tumors with 32,900 deaths annually (Health Commission of Prc, 2019). For now, platinum-based chemotherapy remains the standard first-line treatment for metastatic UC (mUC). However, the prognosis of mUC remains poor, with a median overall survival (OS) of 9–15 months (Bukhari et al., 2018).

Antibodies that block programmed cell death-1 (PD-1) or its ligand 1 (PD-L1) have changed the therapeutic landscape of mUC and are recommended for patients who progress after platinum-based chemotherapy or who are platinum-ineligible (Bellmunt et al., 2017; Elias et al., 2018; Resch et al., 2018; Niglio et al., 2019). However, benefit populations are usually stratified by the positive PD-L1 expression, which has various thresholds. In some circumstances, even with the negative PD-L1 expression, patients still responded to anti–PD-1/PD-L1 treatment (Powles et al., 2018). Therefore, the role of PD-L1 expression in the entire population remains unclear. Furthermore, under the current guideline, the results of PD-L1 expression are not necessary when considering anti–PD-1/PD-L1 therapy (Bukhari et al., 2018), and tumor mutation burden (TMB) and microsatellite instability (MSI-H) are also potential predictors associated with immunotherapy outcomes in melanoma and non–small-cell lung cancer (NSCLC) (Arora et al., 2019). However, the predictive roles of these two biomarkers in mUC are not well established. Furthermore, the inconvenience of these two biomarkers has compromised their advantages for the sequencing process. Therefore, identifying effective and convenient biomarkers for patients who will likely benefit from anti–PD-1/PD-L1 therapy is critical in clinical practice.

The inflammatory process is associated with immunoresistance in patients with malignancies by promoting cancer growth and activating oncogenic signaling (Hanahan and Weinberg, 2011), and pretreatment inflammatory status is associated with clinical outcomes in cancer patients (Laird et al., 2016). Routine peripheral blood parameters, such as absolute neutrophil count, absolute platelet count, and lactate dehydrogenase (LDH) level, serve as potential inflammatory biomarkers that are correlated with poor outcomes in several cancer types (McMillan, 2013). In melanoma and NSCLC, elevated baseline LDH is associated with poor outcome of anti–PD-1 therapy (Ferrucci et al., 2015; Diem et al., 2016; Nakaya et al., 2018; Arora et al., 2019). The neutrophil-to-lymphocyte ratio (NLR), a novel inflammatory biomarker, reportedly has independent prognostic values in patients with melanoma and NSCLC treated with immunotherapy (Arora et al., 2019). In recent years, combining NLR and LDH as a comprehensive predictor has extended their utility in several cancers. The Lung Immune Prognostic Index, based on NLR and LDH, was excellent in predicting the prognosis of patients with advanced NSCLC who received anti–PD-1/PD-L1 therapy (Sorich et al., 2019). Likewise, another similar index, the urothelial immune prognostic index, UIPI, also provided evidence of the utility of combining inflammatory biomarkers in mUC patients receiving PD-1/PD-L1 inhibitors (Banna et al., 2020). However, the optional cut point and the stratification strategy of UIPI are still unclear.

Since accumulating evidence has shown the potential of NLR and LDH in patients with mUC receiving immunotherapy, exploiting the utility of UIPI is an urgent need for stratifying the potential benefited patients. Herein, we reinvestigated the role of UIPI in mUC patients who received anti–PD-1/PD-L1–based therapy and made a modification to test the utility of its capability as an effect and convenient predictor.

## Materials and Methods

### Patients

Patients who had a histologically confirmed diagnosis of mUC (bladder, renal pelvis, or ureter) and were treated with anti–PD-1/PD-L1–based therapy at the Sun Yat-sen University Cancer Center (SYSUCC) between April 2015 and October 2019 were retrospectively identified.

Laboratory results, such as complete blood cell counts, LDH, and albumin levels, at baseline before anti–PD-1/PD-L1–based treatment (within 30 days before the first treatment) were collected from the medical records. Demographic, clinical, and pathological data were also collected. Data for PD-L1 expression were analyzed on tumor cells by immunohistochemistry using 22C3 antibody, and PD-L1 expression >1% was considered PD-L1 positive (Arora et al., 2019). Routine treatment response assessments were performed every two cycles using RECIST (response evaluation criteria in solid tumors) v1.1 (Eisenhauer et al., 2009).

Modified UIPI (mUIPI) was developed based on pretreatment NLR and LDH. The cut point of NLR was in agreement with that in previous reports, which used five at the baseline setting (Ferrucci et al., 2015; Banna et al., 2020), while LDH levels greater than 220U/L were considered to be elevated baseline LDH according to the upper limit of the central laboratory (Tan et al., 2018). Patients were divided into three groups by mUIPI. Good mUIPI, with low NLR and normal LDH; intermediate mUIPI, with high NLR or elevated LDH; and poor mUIPI, with high NLR and elevated LDH.

### Statistical Analysis

The primary endpoint was progression-free survival (PFS), which was defined as the time from the initiation of anti–PD-1/PD-L1–based therapy to the date of disease progression, death, or last date of follow-up. Another endpoint was overall survival (OS), which was defined as the time from the initiation of anti–PD-1/PD-L1–based therapy to the date of mortality. The disease control rate (DCR) was observed as the third endpoint which was defined as complete plus partial response plus stable disease (CR + PR + SD), and progression disease (PD) as non-DCR (Eisenhauer et al., 2009) using the criteria of RECIST version 1.1.

A descriptive analysis was performed. Categorical variables are presented as numbers and percentiles, while medians and ranges are reported for continuous variables. Comparisons between patient characteristics were performed using χ2 or Fisher’s exact test for discrete variables, and the unpaired t-test, the Wilcoxon sign rank test, or analysis of variance for continuous variables. PFS and OS were estimated using the Kaplan–Meier method. Between-group differences in PFS and OS were calculated using the Cox regression model and the log-rank test. Hazard ratios (HRs) and associated 95% confidence intervals (CIs) were calculated using Efron’s method of handling ties. The odds ratio of the DCR was calculated by conditional maximum likelihood estimation (Fisher), and the 95% CI was calculated using exact methods. *p*-values for all two-sided tests are reported, and *p*-values less than 0.05 were considered statistically significant. All statistical analyses were performed using R software version 3.5.3.

## Results

### Patient Characteristics

**FIGURE 1:**
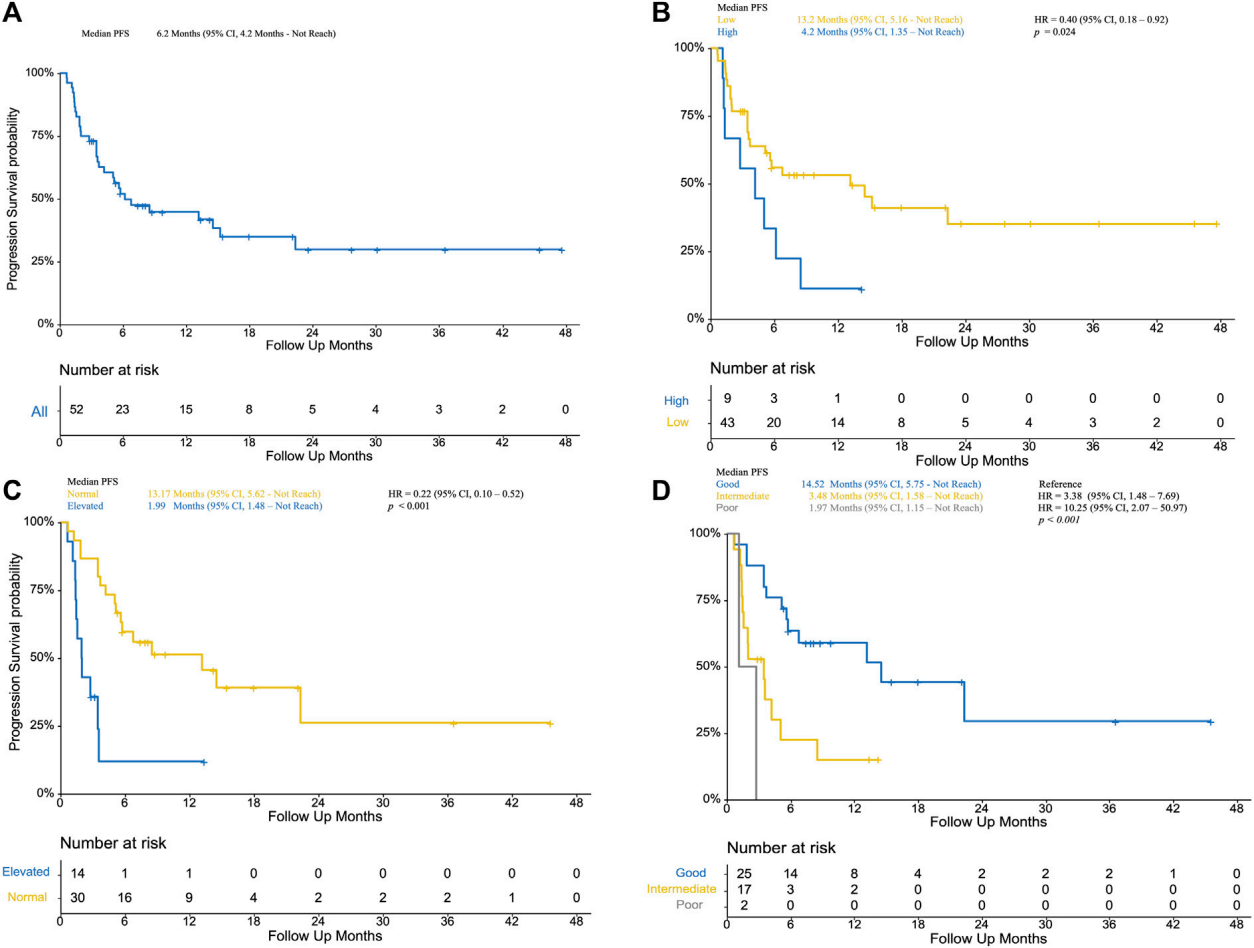
**(A)**: Progression-free survival of included patients; **(B)** Kaplan–Meier curve of low NLR patients (yellow) and high NLR (blue, as reference) patients; **(C)** Kaplan–Meier curve of normal LDH (yellow) and elevated LDH (blue, as reference); **(D)** Kaplan–Meier curve of poor mUIPI (gray), intermediate mUIPI (yellow), and good mUIPI (blue, as reference).

**FIGURE 2:**
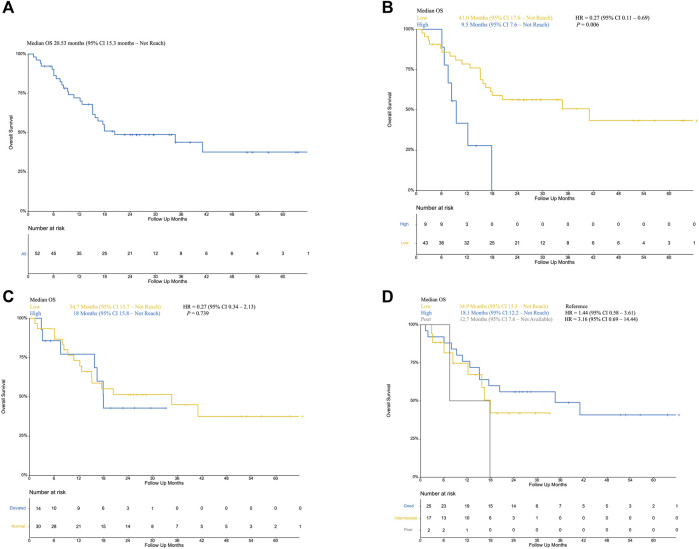
**(A)**: Overall survival of included patients; **(B)** Kaplan–Meier curve of low NLR patients (yellow) and high NLR (blue, as reference) patients; **(C)** Kaplan–Meier curve of normal LDH (yellow) and elevated LDH (blue, as reference); **(D)** Kaplan–Meier curve of poor mUIPI (gray), intermediate mUIPI (yellow), and good mUIPI (blue, as reference).

Fifty-two patients treated with PD-1/PD-L1 inhibitors between April 2015 and October 2019 were included in this study. Baseline characteristics are summarized in Table 1 .With a median follow-up time of 15.5 months (95% CI, 9.8 to 36.6), median PFS was 6.2 months (95% CI, 4.2 to not reached [NR]) (Figure 1A), median OS was 20.53 months (95% CI, 15.3 to not reached [NR]) (Figure 2A). Median patient age was 62 years (range, 34–84). The majority of patients were male (N = 36, 69.2%). Twenty patients (61.54%) were former or current smokers, 32 (61.54%) exhibited liver metastasis, and 10 (19.23%) had an Eastern Cooperative Oncology Group (ECOG) performance status of two or greater. Thirty-one patients (59.61%) exhibited a lower urinary tract primary tumor. In the treatment strategy distribution, 24 patients (46.15%) were first-line setting, and 29 patients (55.76%) were combined with chemotherapy. With 16 available slices of the tumor sample, four patients were considered PD-L1 positive, and PD-L1 expression was not associated with immunotherapy outcomes in this cohort.

**TABLE 1:** Characteristics of included patients.

Characteristic	
Median age, range	62 years, 34–84
Gender	N (%)
Female	16 (30.77)
Male	36 (69.23)
Tobacco use	N (%)
Current/former	20 (38.46)
Never	32 (61.54)
ECOG	N (%)
0–1	42 (80.77)
2–3	10 (19.23)
Primary tumor site	N (%)
Upper tract	21 (40.39)
Low tract	31 (59.61)
Site of metastases	N (%)
Liver	32 (61.54)
Non-liver	20 (38.46)
Regimen	N (%)
Combination	29 (55.77)
Monotherapy	23 (44.23)
Therapy line	N (%)
First line	24 (46.15)
≥ Second line	28 (53.85)
PD-L1 status**^a^**	N (%)
Positive	4 (7.69)
Negative	12 (23.08)
Not available	36 (69.23)
NLR**^a^**	N (%)
High	9 (17.31)
Low	43 (82.69)
LDH**^a^**	N (%)
Elevated	14 (26.92)
Normal	30 (57.69)
Not available	8 (15.38)

a The threshold of PD-L1, NLR, and LDH was 1%, 5, and 220 U/L, respectively.

### NLR and LDH

The median level of NLR was 3.01, ranging from 1.03–7.66. In the predefined context above, forty-three patients were classified as low NLR, which was associated with improved PFS (13.2 VS 4.2 months, *p* = 0.024; HR, 0.40; 95% CI, 0.18–0.92) (Figure 1B) and improved OS (41.0 VS 9.5 months, *p* = 0.006; HR, 0.27; 95% CI, 0.11–0.69) (Figure 2B) but was not associated with higher DCR (odds ratio [OR], DCR VS non-DCR, 1.46, 95% CI, 0.26–6.89, *p* = 0.69) (Table 2). As for the LDH level, thirty patients had normal LDH, which was associated with improved PFS (13.17 VS 1.99 months, *p* < 0.001; HR, 0.22; 95% CI, 0.08–0.55, *p* < 0.001) (Figure 1C) and higher DCR (OR, DCR VS non-DCR, 10.84, 95% CI, 2.08–70.67, *p* < 0.001) (Table 2) but was not statistically associated with improved OS (34.7 VS 18 months, *p* = 0.739; HR, 0.27; 95% CI, 0.34–2.13) (Figure 2C).

**TABLE 2:** Efficacy (DCR) analysis of included patients.

Characteristic	OR	95% CI	*p*
Gender			
Female	1	Reference	—
Male	1.37	0.34–5.06	0.738
Tobacco use			
Current/former	1	Reference	—
Never	1.9	0.53–6.88	0.299
ECOG			
0–1	1	Reference	—
2–3	0.47	0.11–2.26	0.428
Primary tumor site			
Upper tract	1	Reference	—
Low tract	0.77	0.2–2.76	0.677
Site of metastases			
Liver	1	Reference	—
Non-liver	1.16	0.32–4.54	0.805
Regimen			
Combination	1	Reference	—
Monotherapy	0.5	0.13–1.75	0.255
Therapy line			
First line	1	Reference	—
≥ Second line	0.37	0.09–1.36	0.122
NLR			
High	1	Reference	—
Low	1.47	0.27–6.89	0.688
LDH			
Elevated	1	Reference	—
Normal	10.62	2.45–56.39	<0.001
mUIPI groups			
Good	1	Reference	—
Intermediate	0.13	0.02–0.58	0.004
Poor	0.15	0.003–7.04	0.296

In a multivariate analysis, among variables with *p*-values less than 0.1 or with clinical meaning, normal LDH (HR, 0.19; 95% CI, 0.07–0.48, *p* < 0.001) and first-line treatment (HR, 0.35; 95% CI, 0.14–0.91, *p* = 0.03) were independently associated with improved PFS, while low NLR (HR, 0.49; 95% CI, 0.18–1.36, *p* = 0.17) was not. However, the low NLR was an independent predictor in OS (HR, 0.30; 95% CI, 0.12–0.79, *p* = 0.014).

In the subgroup analysis, NLR and LDH had performed well in predicting clinical outcomes of patients treated with anti–PD-1/PD-L1 monotherapy in PFS and OS. (low NLR, HR, 0.343 for PFS, 0.128 for OS; normal LDH, HR, 0.042 for PFS, 0.194 for OS) (Supplementary Table1). And low NLR was also associated with improved survival outcomes in patients with liver metastases (Supplementary Table1).

### Modified Urothelium Immune Prognostic Index (mUIPI)

Pretreatment NLR and LDH were combined with the calculation of mUIPI. Eight patients without baseline LDH were excluded from the UIPI analysis. Among 44 evaluable patients, 25 (56.81%) had good mUIPI, 17 (38.64%) had intermediate mUIPI, and 2 (4.55%) had poor mUIPI (Table 3).

**TABLE 3:** Characteristics of patients included in the mUIPI analysis.

Characteristic	Good (25), n (%)	Intermediate (17), n (%)	Poor (2), n (%)	*p*
Gender	—	—	—	0.748
Female	7 (28)	4 (23.53)	1 (50)	—
Male	18 (72)	13 (76.47)	1 (50)	—
Tobacco use	—	—	—	0.514
Current/former	8 (32)	8 (47.06)	1 (50)	—
Never	17 (68)	9 (52.94)	1 (50)	—
ECOG				0.834
0–1	19 (76)	14 (82.35)	2 (100)	—
2–3	6 (24)	3 (17.65)	0 (0)	—
Primary tumor site	—	—	—	1
Upper tract	10 (40)	7 (41.18)	1 (50)	—
Low tract	15 (60)	10 (58.82)	1 (50)	—
Site of metastases	—	—	—	0.591
Non-liver	10 (40)	8 (47.06)	0 (0)	—
Liver	15 (60)	9 (52.94)	2 (100)	—
Therapy line	—	—	—	0.203
>=Second line	12 (48)	12 (70.59)	2 (100)	—
First line	13 (52)	5 (29.41)	0 (0)	—
Regimen	—	—	—	0.251
Combination	16 (64)	10 (58.82)	0 (0)	—
Monotherapy	9 (36)	7 (41.18)	2 (100)	—
PD-L1 status**^a^**	—	—	—	
Positive	3 (34)	1 (86)	0 (0)	0.585
Negative	6 (66)	6 (14)	0 (0)	—

a PD-L1 analysis was based on 16 available samples.

The median PFS was 1.97 months (95% CI, 1.15 month to NR) vs. 3.48 months (95% CI, 1.58 months to NR) vs. 14.52 months (95% CI, 5.75 months to NR) for the poor, intermediate, and good mUIPI groups, respectively (*p* < 0.001) (Figure 1D). The median OS of each group was 12.7, 18.1, and 34.9 months, respectively. (Figure 2D). A good mUIPI was associated with higher DCR compared to intermediate and poor mUIPI (OR, non-DCR VS DCR, 0.13, 95% CI, 0.02–0.58; OR, 0.15, 95% CI, 0.003–7.04, respectively) (Table 2). In a multivariate analysis, intermediate and poor mUIPI groups were independently associated with shorter PFS (HR, 3.38, 95% CI, 1.48–7.69; HR, 10.25, 95% CI, 2.03–20.97, compared to good mUIPI, respectively). Therapy line was also associated with improved PFS (first-line VS ≥ second-line, HR, 0.39, 95% CI, 0.15–0.97) (Table 4). However, mUIPI failed to cross the statistical boundary in the cox regression analysis (Table 5).

**TABLE 4:** Cox proportional hazard regression model of PFS on included patients.

Characteristic	Univariate Cox regression			Multivariate Cox regression		
	HR	CI	*P*	HR	CI	*P*
Gender						
Female	Reference	Reference	—	—	—	—
Male	0.78	0.37–1.63	0.511	—	—	—
Tobacco use						
Current/former	Reference	Reference	—	—	—	—
Never	0.80	0.39–1.64	0.550	—	—	—
ECOG						
0–1	Reference	Reference	—	Reference	Reference	—
2–3	1.71	0.72–4.03	0.222	2.74	0.99–7.613	0.053
Primary tumor site						
Upper tract	Reference	Reference	—	—	—	—
Low tract	0.52	0.40–1.69	0.597	—	—	—
Site of metastases						
Non-liver	Reference	Reference	—	Reference	Reference	—
Liver	1.45	0.67–3.15	0.352	1.60	0.57–4.445	0.369
Therapy line						
≥ Second line	Reference	Reference	—	Reference	Reference	—
First line	0.41	0.2–0.87	0.020	0.39	0.15–0.973	0.044
PD-L1 status						
Negative	Reference	Reference	—	—	—	—
Positive	0.836	0.22–3.15	0.791	—	—	—
Regimen						
Combination	Reference	Reference		Reference	Reference	—
Monotherapy	1.16	0.57–2.36	0.680	1.25	0.47–3.327	0.660
mUIPI groups						
Good	Reference	Reference	—	Reference	Reference	—
Intermediate	3.38	1.48–7.69	0.004	4.15	1.69–10.188	0.002
Poor	10.25	2.06–50.97	0.004	7.80	1.29–47.15	0.025
NLR						
High	Reference	Reference	—	—	—	—
Low	0.40	0.18–0.92	0.030	—	—	—
LDH						
Elevated	Reference	Reference	—	—	—	—
Normal	0.22	0.1–0.52	0.001	—	—	—

**TABLE 5:** Cox proportional hazard regression model of OS on included patients.

Characteristic	Univariate Cox regression			Multivariate Cox regression		
	HR	CI	*P*	HR	CI	*P*
Gender						
Female	Reference	Reference	—	—	—	—
Male	0.64	0.29–1.38	0.250	—	—	—
Tobacco use						
Current/former	Reference	Reference	—	—	—	—
Never	1.43	0.62–3.29	0.402	—	—	—
ECOG						
0–1	Reference	Reference	—	Reference	Reference	—
2–3	2.08	0.83–5.23	0.120	2.58	1.00–6.67	0.049
Primary tumor site						
Upper tract	Reference	Reference	—	—	—	—
Low tract	1.09	0.51–2.33	0.831	—	—	—
Site of metastases						
Non-liver	Reference	Reference	—	—	—	—
Liver	1.14	0.51–2.54	0.745	—	—	—
Therapy line						
≥ Second line	Reference	Reference	—	Reference	Reference	—
First line	0.80	0.37–1.73	0.577	0.81	0.37–1.77	0.600
PD-L1 status						
Negative	Reference	Reference	—	—	—	—
Positive	0.25	0.03–2.03	0.195	—	—	—
Regimen						
Combination	Reference	Reference	—	—	—	—
Monotherapy	0.86	0.40–1.86	0.708	—	—	—
mUIPI groups						
Good	Reference	Reference	—	—	—	—
Intermediate	1.44	0.58–3.61	0.433	—	—	—
Poor	3.16	0.69–14.44	0.139	—	—	—
NLR						
High	Reference	Reference		Reference	Reference	—
Low	0.27	0.11–0.69	0.006	0.24	0.09–0.63	0.004
LDH						
Elevated	Reference	Reference	—	—	—	—
Normal	0.86	0.34–2.13	0.739	—	—	—

**FIGURE 3:**
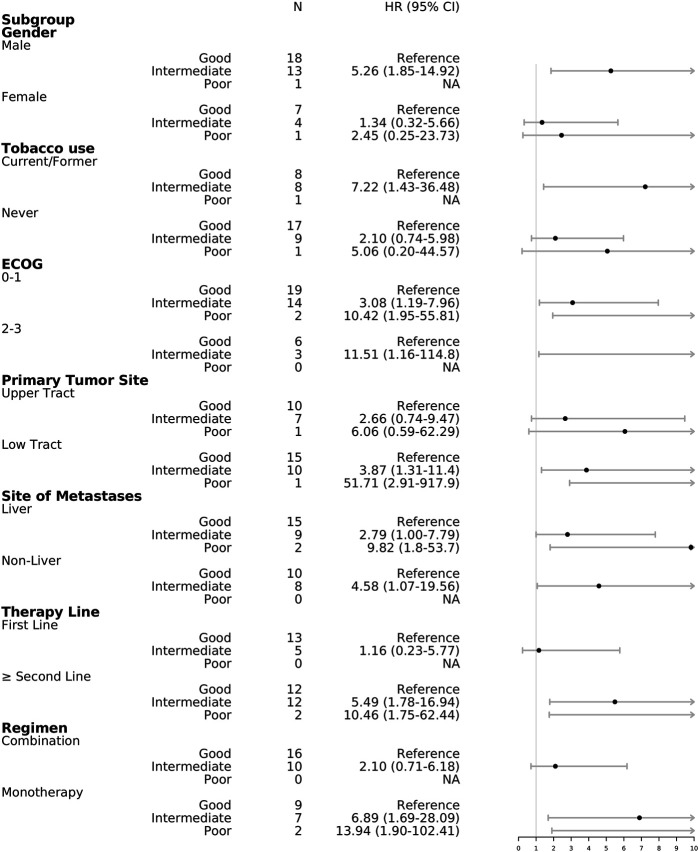
Forest plot of hazard ratios in the subgroup analysis comparing progression-free survival of mUIPI.

**FIGURE 4:**
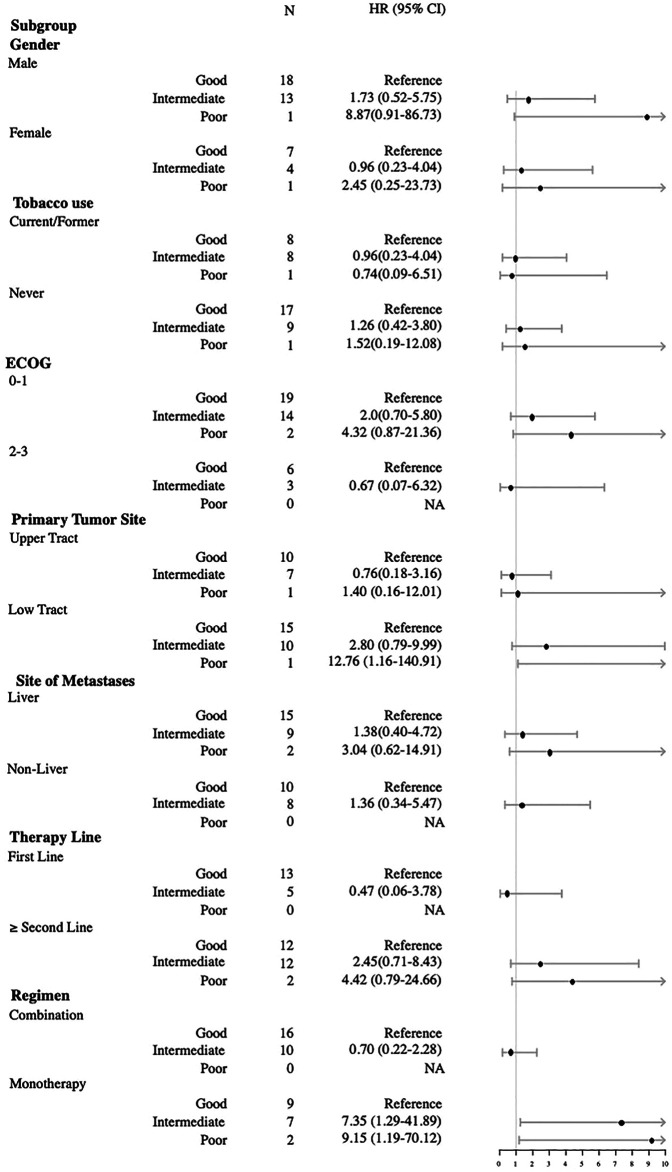
Forest plot of hazard ratios in the subgroup analysis comparing overall survival of mUIPI.

In the subgroup analysis, good mUIPI was associated with improved PFS in male, current/former smoker, low-tract primary tumor, ECOG of 0–1 and two to three, liver and non-liver metastases, non–first-line treatment, and monotherapy subgroups (Figure 3). Meanwhile, good mUIPI was associated with higher DCR in male, low-tract primary tumor, liver metastases, non–first-line treatment, and monotherapy subgroups. As for the subgroup analysis of OS, good mUIPI was associated with prolonged OS in the subgroup of monotherapy (Figure 4).

In the anti–PD-1/PD-L1 monotherapy subgroup, good mUIPI was associated with improved PFS, OS, as well as higher DCR. Median PFS was 1.97 months (95% CI, 1.15 month to NR) vs. 2.00 months (95% CI, 1.48 months to NR) vs. 22.34 months (95% CI, 5.94 months to NR) for poor, intermediate, and good mUIPI groups, respectively (*p* < 0.001), and median OS was 12.7 months (95% CI, 7.6 months to NR) vs. 15.8 months (95% CI, 8.4 months to NR) vs. NR (95% CI, 41.0 months to NR), respectively (*p* = 0.017). Furthermore, patients with good mUIPI had a higher DCR (good vs. intermediate, OR, 14.52, 95% CI, 1.34–523.98; good vs. poor, OR, 6.00, 95% CI, 0.10–342.93). In the multivariate analysis, good mUIPI was independently associated with improved PFS (intermediate vs. good, HR, 13.74, 95% CI, 1.46–129.08; poor vs. good, HR, 8.89, 95% CI, 1.09–72.57) and OS (intermediate vs. good, HR, 7.95, 95% CI, 1.14–55.61; poor vs. good, HR, 8.57, 95% CI, 1.11–66.26), whereas in the combined therapy subgroup, treatment outcomes were not statistically significantly correlated with mUIPI.

## Discussion

In the present study, we reinvestigated the utility of UIPI and developed the modified UIPI, based on a definite cut point of pretreatment NLR and LDH, which was significantly associated with PFS and short-term treatment response in patients with mUC treated with anti–PD-1/PD-L1–based therapy, exerting utility in several subgroups. Based on mUIPI, patients were stratified into three groups: good, intermediate, and poor mUIPI. Specifically, good mUIPI was significantly associated with prolonged PFS and higher DCR, indicating that it might represent a predictor for identifying populations that will benefit from anti–PD-1/PD-L1–based treatment.

In patients with malignancies, inflammatory status was closely correlated with poor clinical outcomes (Laird et al., 2016). With the advent of immune checkpoint inhibitors (ICIs), especially PD-1/PD-L1 inhibitors, inflammatory status is also associated with immunotherapy benefit (Arora et al., 2019). Several potential inflammatory biomarkers, including elevated neutrophils, lymphocytes, platelets, and LDH, are associated with poor outcomes in cancer. NLR, a novel parameter, is a well-known prognostic factor in cancer patients with late-stage melanoma or NSCLC treated with ICIs. In a retrospective cohort of 187 metastatic melanoma patients treated with ipilimumab, pretreatment NLR less than five was associated with improved survival (Ferrucci et al., 2015). In another retrospective cohort of 101 advanced NSCLC patients treated with nivolumab, high NLR 2 and 4 weeks after treatment was associated with poor treatment response and PFS (Nakaya et al., 2018). dNLR has also been investigated as a useful predictor of clinical outcomes in various cancer types, including melanoma, pancreas, and bladder cancer. In a retrospective study of 720 melanoma patients, dNLR of three or greater was independently associated with worse survival in patients treated with ipilimumab (Ferrucci et al., 2018). However, in a cohort of 123 muscle-invasive bladder cancer patients, elevated dNLR (>2.21) was correlated with nonresponse to preoperative chemotherapy (OR 2.70, 95% CI, 1.15–6.38) but was not statistically significantly associated with poor PFS or OS (van Kessel et al., 2016). In the present study, we found that NLR of five or greater was significantly correlated with inferior PFS and OS in mUC patients treated with anti–PD-1/PD-L1–based therapy. Furthermore, in the multivariate analysis, we reveal that high NLR was independently associated with inferior OS although it failed to cross the statistical boundary in PFS.

The serum LDH level is a standardized and convenient inflammatory biomarker in patients with cancer that is widely studied in the era of chemotherapy (Petrelli et al., 2015). Recently, elevated baseline LDH levels were been found to be associated with poor survival and a decreased response rate in patients treated with ICIs (Arora et al., 2019). In a cohort of 668 patients with upper tract urothelial carcinoma, preoperative elevated LDH was found to be correlated with worse OS in patients with localized disease (Tan et al., 2018). In the present study, we demonstrated that LDH represents an independent prognostic factor in patients with mUC treated with anti–PD-1/PD-L1–based therapy because normal LDH was associated with significantly improved PFS and treatment response. Therefore, LDH levels could be a useful and convenient predictor of response to immunotherapy.

Combining biomarkers, such as CII and LIPI, exploited the potential of NLR and LDH in colorectal and lung cancer (Casadei-Gardini et al., 2019; Sorich et al., 2019). In a similar previous study, UIPI was able to stratify benefited mUC patients treated with immunotherapy. However, the cut point and optional classification of UIPI remained inconclusive. In the present study, we modified the UIPI based on a definite cut point of pretreatment NLR and LDH. The mUIPI was able to identify three populations and showed that good mUIPI was significantly associated with superior immunotherapy outcomes. Furthermore, in patients treated with PD-1/PD-L1 inhibitors alone, mUIPI was also capable to identify a potential benefit subgroup, and it was useful for physicians to prescribe immunotherapy since patients with mUC usually suffer from renal function impairment, which is a contraindication for various cytotoxic drugs. Nevertheless, mUIPI is easy to extract from the routine blood parameters, while other prognostic factors, such as PD-L1 expression, are not definitively correlated with treatment outcomes in urothelial cancer and are not mandatory for prescribing the immunotherapy under the current guidelines. In addition, TMB, which was associated with immunotherapy outcomes in urothelial cancer, is limited by a complicated and expensive sequencing process. Therefore, UIPI, with its convenient generation and reliability, could be used to identify patients who might benefit from anti–PD-1/PD-L1–based therapy.

Combining PD-1/PD-L1 inhibitors with conventional chemotherapy is a recent and intriguing topic. IMvigor 130 was designed to investigate this modality. With the recent release of preliminary results, the study demonstrated that adding atezolizumab to platinum-based chemotherapy (PBC) improved PFS over PBC alone (Galsky et al., 2020). Regrettably, due to the PFS data of atezolizumab being absent, whether atezolizumab plus PBC provides a PFS advantage over atezolizumab alone in untreated mUC patients remained unknown. Meanwhile, in our study, combination therapy was not superior to monotherapy. However, the results might be comprised since the precisions of the combination therapy in our study consisted of various chemotherapy regimens rather than single PBC. In addition, some patients in our study were heavily treated, and some patients had a worse performance status and vulnerable renal function, which might have contributed to this dubious result. With ongoing treatment, cytotoxic drugs might further exacerbate renal malfunction, which might result in a worse prognosis for mUC patients. Therefore, more trials are needed to complete the picture and to identify populations who will benefit most from immunotherapy.

The nature of retrospective data is the primary limitation of our study, including missing clinical and laboratory data, as well as a high rate of patients with an unknown PD-L1 status. Another limitation was the small sample size of the cohort, which might lead to an underpower of this study, thus compromising the predicting strength of mUIPI. Also, the heterogeneity of chemotherapy regimens in the combination groups compromised the results of this study.

In summary, we demonstrated that mUIPI, based on pretreatment NLR and LDH levels, is correlated with treatment outcomes of anti–PD-1/PD-L1–based therapy in patients with mUC.

## Data Availability

The raw data supporting the conclusion of this article will be made available by the authors, without undue reservation. The key raw data has been deposited onto the Research Data Deposit public platform (www.researchdata.org.cn), with the approval RDD number as RDDA2021002042.

## Abbreviations

CII, colon inflammatory index; DCR, disease control rate; dNLR, derived NLR; ECOG PS, ECOG performance-status score; HR, hazard ratio; ICI, immune checkpoint inhibitor; LDH, lactate dehydrogenase; mUC, metastatic urothelial carcinoma; MSI-H, microsatellite instability; NLR, neutrophil-to-lymphocyte ratio; NSCLC, non–small-cell lung cancer; OR, odds ratio; OS, overall survival; PFS, progression-free survival; PD-1, programmed cell death 1; PD-L1, programmed cell death 1 ligand 1; TMB, tumor mutation burden; UIPI, urothelium immune prognostic index.
