# Supplementary material for: Role of a Modified Urothelium Immune Prognostic Index in Patients With Metastatic Urothelial Carcinoma Treated With Anti–PD-1/PD-L1–Based Therapy
Source: Front Mol Biosci. 2021 Aug 12;8:621883. doi: 10.3389/fmolb.2021.621883 (PMC8387676; doi:10.3389/fmolb.2021.621883)
Supplement: Supplementary file 1 [file Table1.DOCX]

| sTable1: The subgroup survival analysis of NLR and LDH | | | | | | | | | | | |
| --- | --- | --- | --- | --- | --- | --- | --- | --- | --- | --- | --- |
|  |  |  | NLR | | | |  | LDH | | | |
|  |  |  | PFS | | OS | |  | PFS | | OS | |
| **Subgroup** | Arm | N | HR | 95% CI | HR | 95% CI | Arm | HR | 95% CI | HR | 95% CI |
| **Gender** |  |  |  |  |  |  |  |  |  |  |  |
| Male | Low Versus High | 36 | 0.507 | 0.186-1.381 | 0.235 | 0.062-0.885 | Normal Versus Elevated | 0.265 | 0.109-0.644 | 0.731 | 0.219-2.439 |
| **Female** | Low Versus High | 16 | 0.322 | 0.076-1.368 | 0.434 | 0.111-1.697 | Normal Versus Elevated | 0.808 | 0.205-3.181 | 1.437 | 0.351-5.887 |
| Age |  |  |  |  |  |  |  |  |  |  |  |
| <65 | Low Versus High | 30 | 0.442 | 0.168-1.165 | 0.362 | 0.121-1.081 | Normal Versus Elevated | 0.405 | 0.172-0.954 | 0.934 | 0.344-2.541 |
| ≥ 65 | Low Versus High | 22 | 0.478 | 0.101-2.266 | 0.186 | 0.03-1.144 | Normal Versus Elevated | 0.374 | 0.091-1.533 | 0.893 | 0.092-8.627 |
| **Tobacco Use** |  |  |  |  |  |  |  |  |  |  |  |
| Current/Former | Low Versus High | 20 | 0.292 | 0.085-1.006 | NA | NA | Normal Versus Elevated | 0.192 | 0.053-0.692 | 0.546 | 0.11-2.71 |
| Never | Low Versus High | 32 | 0.491 | 0.16-1.502 | 0.451 | 0.142-1.428 | Normal Versus Elevated | 0.548 | 0.195-1.539 | 0.984 | 0.312-3.103 |
| **ECOG** |  |  |  |  |  |  |  |  |  |  |  |
| 0-1 | Low Versus High | 42 | 0.404 | 0.165-0.984 | 0.206 | 0.071-0.6 | Normal Versus Elevated | 0.458 | 0.201-1.04 | 0.723 | 0.262-1.997 |
| 2-3 | Low Versus High | 10 | NA | NA | 0.266 | 0.024-2.955 | Normal Versus Elevated | 0.087 | 0.009-0.866 | 1.458 | 0.158-13.429 |
| **Site of metastases** |  |  |  |  |  |  |  |  |  |  |  |
| Non-Liver | Low Versus High | 20 | 0.542 | 0.117-2.522 | 0.679 | 0.079-5.826 | Normal Versus Elevated | 0.496 | 0.16-1.542 | 0.873 | 0.208-3.668 |
| Liver | Low Versus High | 32 | 0.353 | 0.133-0.938 | 0.211 | 0.071-0.621 | Normal Versus Elevated | 0.315 | 0.121-0.819 | 0.864 | 0.266-2.814 |
| **Therapy line** |  |  |  |  |  |  |  |  |  |  |  |
| ≥ Second Line | Low Versus High | 28 | 0.514 | 0.183-1.446 | 0.316 | 0.093-1.07 | Normal Versus Elevated | 0.274 | 0.11-0.685 | 0.569 | 0.19-1.698 |
| First Line | Low Versus High | 24 | 0.347 | 0.093-1.294 | 0.248 | 0.058-1.059 | Normal Versus Elevated | 0.94 | 0.202-4.37 | NA | NA |
| **Regimen** |  |  |  |  |  |  |  |  |  |  |  |
| Combination | Low Versus High | 29 | 0.669 | 0.153-2.938 | 0.435 | 0.092-2.059 | Normal Versus Elevated | 0.788 | 0.282-2.196 | 2.527 | 0.558-11.444 |
| Monotherapy | Low Versus High | 23 | 0.343 | 0.118-1.000 | 0.128 | 0.032-0.516 | Normal Versus Elevated | 0.042 | 0.005-0.355 | 0.194 | 0.045-0.835 |
